# Rotating Hinge Knee Arthroplasty for Charcot Joints of the Knees in Patients With Charcot–Marie–Tooth Disease: A Report of Two Cases

**DOI:** 10.7759/cureus.63154

**Published:** 2024-06-25

**Authors:** Yutaka Ehara, Naoki Nakano, Koji Takayama, Yuichi Kuroda, Shingo Hashimoto, Shinya Hayashi, Takehiko Matsushita, Takahiro Niikura, Ryosuke Kuroda, Tomoyuki Matsumoto

**Affiliations:** 1 Department of Orthopaedic Surgery, Kobe University Graduate School of Medicine, Kobe, JPN

**Keywords:** charcot-marie-tooth disease, neurogenic, arthropathy, replacement, unstable knee

## Abstract

We report two cases wherein rotating hinge knee (RHK) arthroplasty was performed for Charcot joints that developed secondary to Charcot-Marie-Tooth disease (CMT).

Case 1 was of a 74-year-old woman with CMT. She presented with muscle weakness and sensory disturbances of the distal lower limbs, deformity, and significant medial instability of the bilateral knees. She was then diagnosed with Charcot joints of the knees secondary to CMT, which were treated with RHK arthroplasty. Five years postoperatively, there was no instability, and she was able to stand unassisted without pain. Case 2 was a 90-year-old woman with CMT who presented with muscle weakness and sensory disturbances of the distal lower limbs, deformity, and significant medial instability of the bilateral knees. She was then diagnosed with Charcot joints of the knees secondary to CMT, which were also treated with RHK arthroplasty. One year postoperatively, there was no instability, and she was able to walk smoothly using a walker. These clinical cases indicate that RHK arthroplasty can be a good therapeutic option for Charcot joints of the knees in patients with CMT.

## Introduction

Charcot-Marie-Tooth disease (CMT) is the most common type of hereditary motor and sensory neuropathy, and the most common inheritance pattern is autosomal dominant. CMT typically develops and progresses slowly, and results in an unsteady walk. CMT leads to a type of neurogenic arthropathy called Charcot joint [1]. Its etiology is unclear, and a variety of central or peripheral nerve-damaging diseases can cause similar damage. CMT causes Charcot joints of lower limbs, particularly in those of the ankles and toes. While foot deformities are the most common feature, knee deformities are rare [2,3]. Although total knee arthroplasty (TKA) used to be contraindicated for Charcot joints [4,5], it has recently been regarded as a treatment option for Charcot joints provided the underlying disease has been treated [6-9]. However, only a few reports have been published on the treatment of Charcot joints of the knees in patients with CMT [10]. We report two cases of Charcot joints caused by CMT that were successfully treated with rotating hinge knee (RHK) arthroplasty. We also review the relevant literature concerning the treatment of Charcot joints secondary to CMT.

## Case presentation

Case 1

A 74-year-old woman with a medical history of hepatitis C presented with complaints of progressive deformity and instability of both knees. She had no history of diabetes mellitus or neurosyphilis. The patient developed progressive weakness of the distal muscle of the upper limbs over 20 years. She started experiencing pain in both knees, which was treated conservatively at a nearby clinic. She also developed lower limb muscle weakness. A nerve conduction study (NCS) showed reduced compound muscle action potential (CMAP) amplitude, slowed conduction velocity, extended distal latency, and an absence of sensory nerve action potential amplitude. Sural nerve biopsies showed onion bulb formation and decreased myelinated nerve fibers. Two years before presenting at our institution, the presence of a PMP22 duplication suggested that she had CMT type 1A (CMT1A) [11]. Her right knee showed significant instability, and she found it difficult to stand and was unable to walk. Therefore, she was referred to our hospital for further treatment.

A physical examination revealed an inability to stand and walk unassisted, with a valgus deformity, swelling, and effusion of the right knee. There were no signs of ulceration. The range of motion of the knee joint was 130 degrees in flexion and 0 degrees in extension, and there was severe medial instability. The distal lower limbs showed muscle weakness, numbness, and hypopallesthesia. Both ankle joints showed varus deformity.

**Figure 1:**
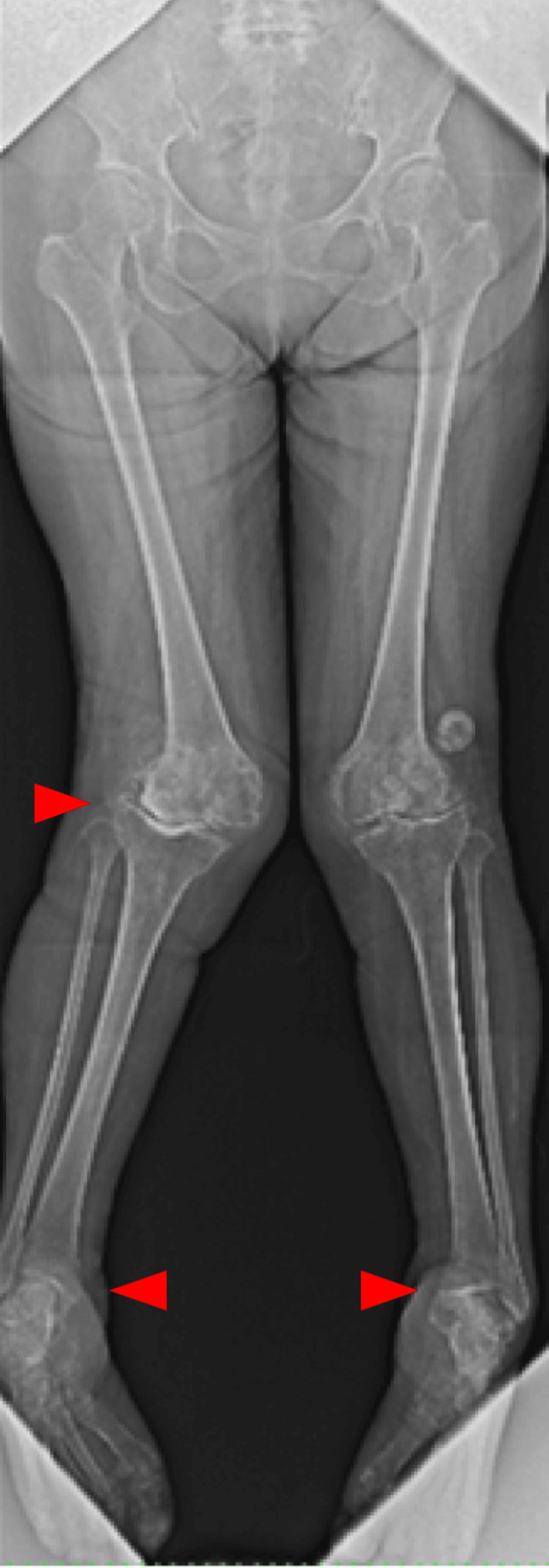
Anteroposterior long-leg lying image of both legs

**Figure 2:**
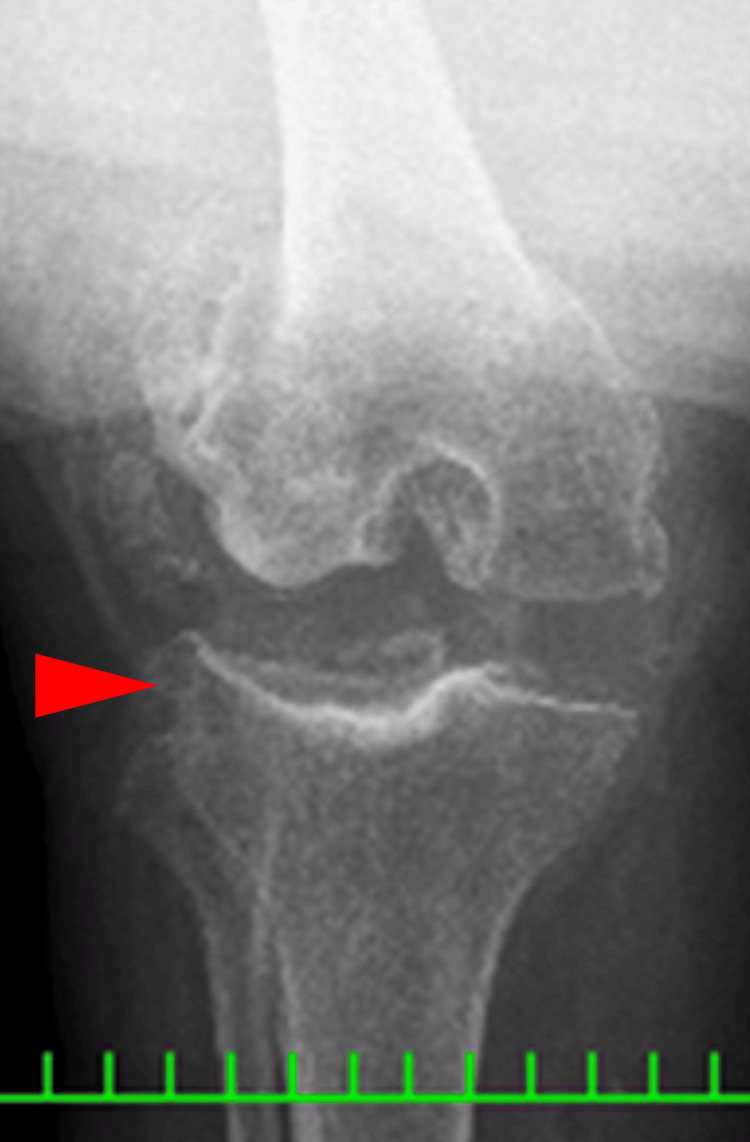
Anteroposterior one-leg lying image of the right knee

**Figure 3:**
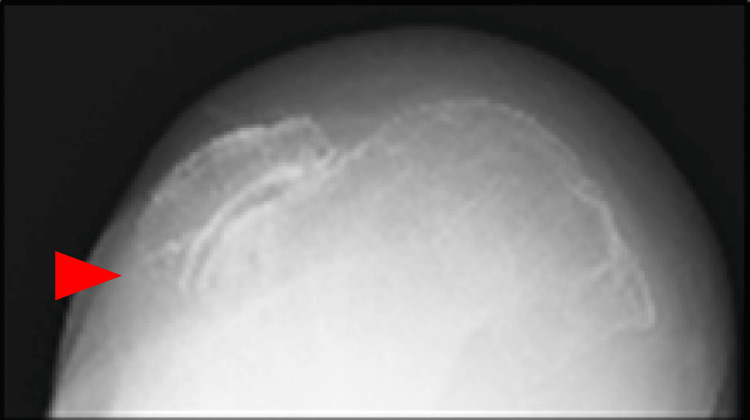
Skyline view of the patella at 30 degrees of knee flexion

Coagulation and syphilis tests were normal; however, reduced nerve conduction velocity was noted. A radiograph showed an obvious valgus deformity and destruction of the tibiofemoral joint: the Hip-knee-ankle angle (HKA) of the right knee was 26 degrees, the lateral distal femoral angle (LDFA) of the right knee was 76 degrees, and the medial proximal tibial angle (MPTA) of the right knee was 105 degrees. The Knee Society Score (KSS) clinical score was 13 and the KSS functional score was -4. A lateral shift of the patella and hypoplasia of the femoral trochlea were also observed. Both ankle joints were painless but showed varus deformity (Figures 1-3).

She was then diagnosed with Charcot joints secondary to CMT based on her clinical history, physical examination, and imaging findings and was treated with TKA using a NexGen® Rotating Hinge Knee implant (Zimmer Biomet, Warsaw, IN, USA) following the standard medial parapatellar approach. Defects of the lateral tibial condyle and patella were limited; thus, no augmentation or patellar resurfacing was performed.

**Figure 4:**
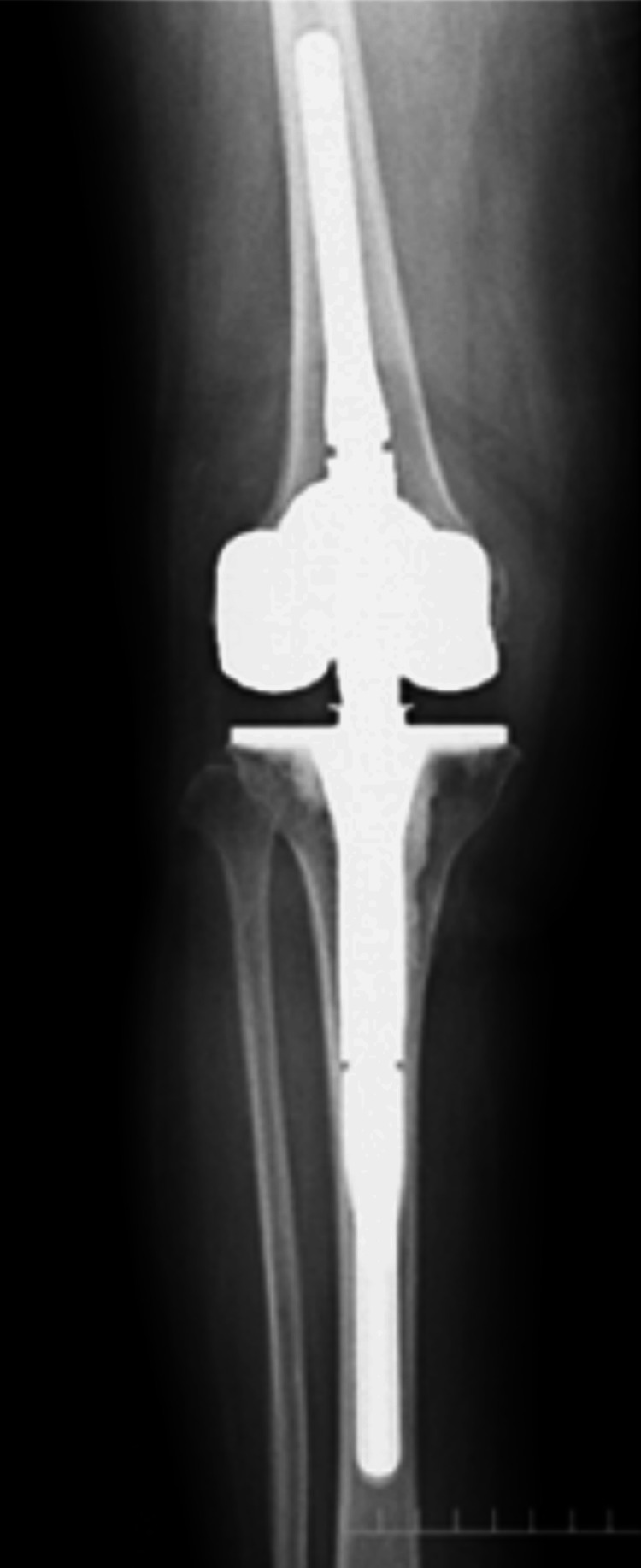
Anteroposterior one-leg standing image of the right knee

**Figure 5:**
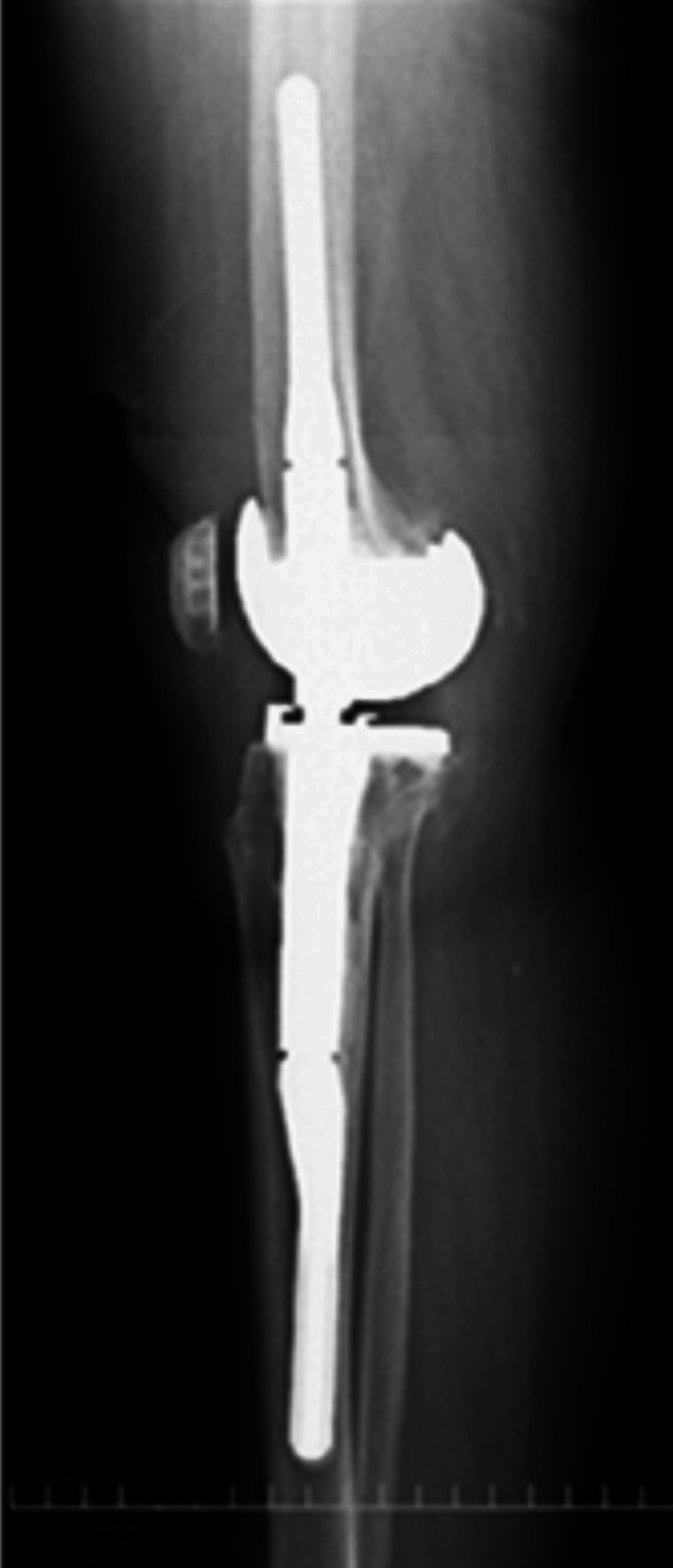
Lateral one-leg standing image of the right knee

**Figure 6:**
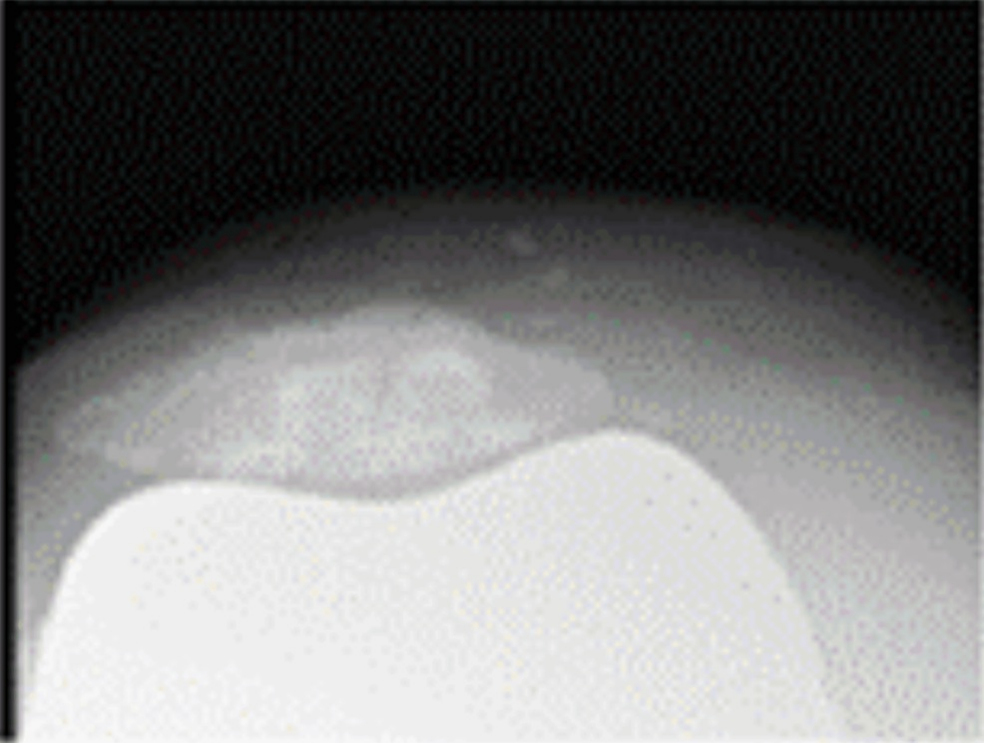
Skyline view of the patella at 30 degrees of knee flexion

One year postoperatively, the right knee range of motion was 125 degrees in flexion and 0 degrees in extension. There was no medial instability, and the patient was able to stand and transfer to a wheelchair without pain and assistance. X-rays showed an HKA of 1, LDFA of 87, MPTA of 88 degrees for the right knee, and no loosening or sinking (Figures 4-6). The KSS clinical score was 72 and KSS functional score was -1. Five years postoperatively, the right knee range of motion was 130 degrees in flexion and 0 degrees in extension. There was no medial instability, and the patient was able to stand and get in a wheelchair without pain and assistance.

Case 2

A 90-year-old woman with a history of hypertension presented with complaints of progressive instability and deformity of both knees. There was no history of diabetes mellitus or neurosyphilis. The patient developed progressive muscle weakness in the distal upper limbs over 6 years. An NCS showed reduced CMAP amplitude, decreased conduction velocity, extended distal latency, and an absence of sensory nerve action potential amplitude. Nerve biopsies showed onion bulb formation and decreased myelinated nerve fibers. The presence of a PMP22 duplication suggested CMT1A. She had pain in both knees, which was treated conservatively at a nearby clinic. She was diagnosed with CMT after genetic testing. One year before presenting at our institution, her left knee became unstable; hence, she was unable to walk. She was referred to our hospital for further treatment afterward.

A physical examination revealed valgus deformity, swelling, and effusion of the left knee. There were no ulcerations. The range of motion of the knee joint was 100 degrees in flexion and 0 degrees in extension, and there was severe medial instability. The patient was barely able to walk with the help of a walker. She did not have weakness of the extensor hallucis longus but had numbness and hypopallesthesia of the distal lower limbs.

**Figure 7:**
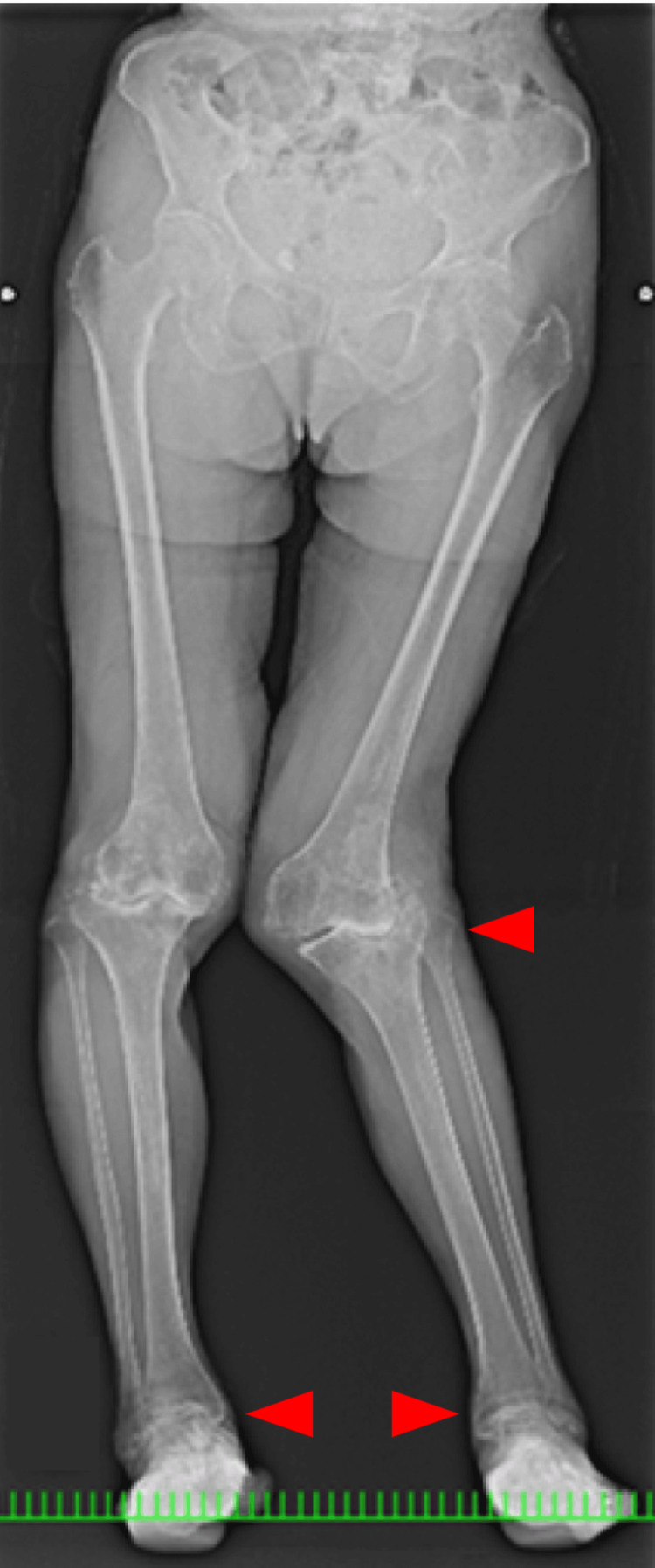
Anteroposterior long-leg standing image of both legs

**Figure 8:**
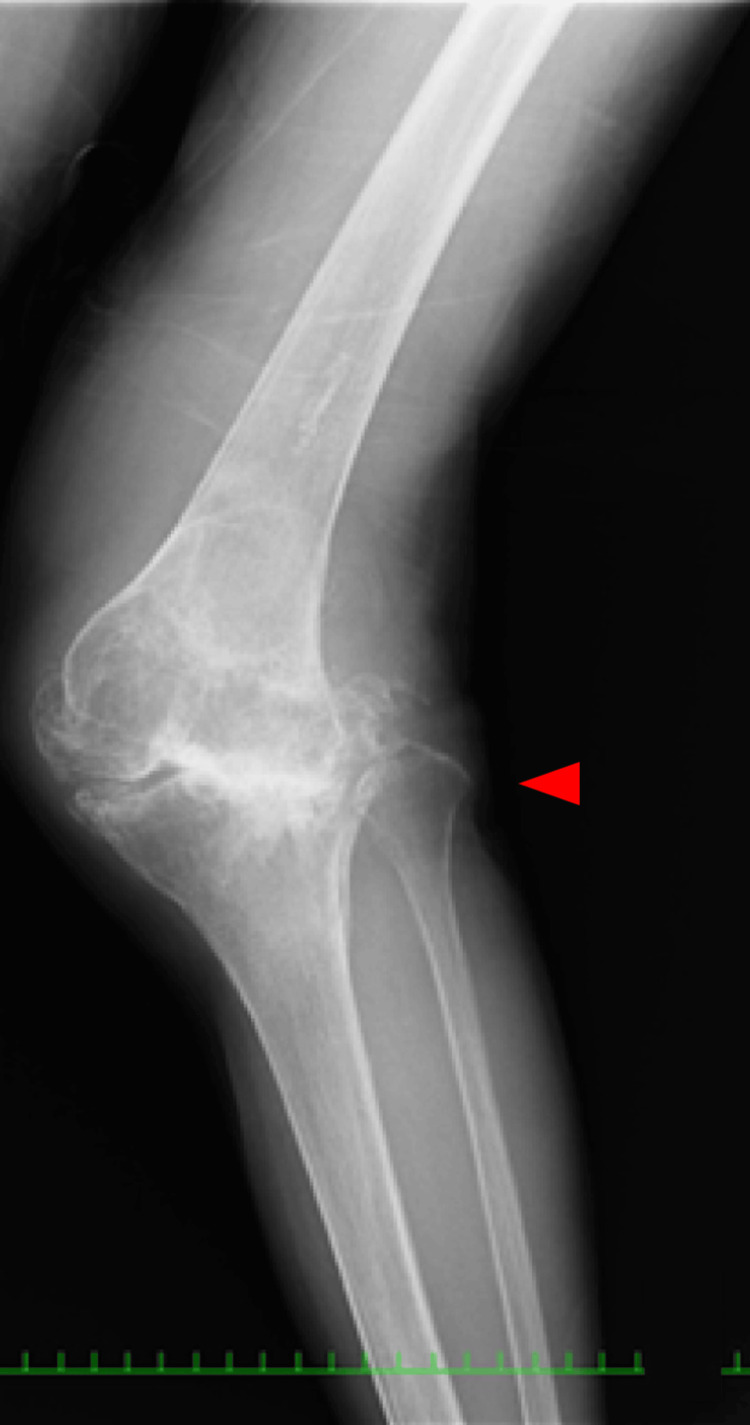
Anteroposterior one-leg standing image of the left knee

**Figure 9:**
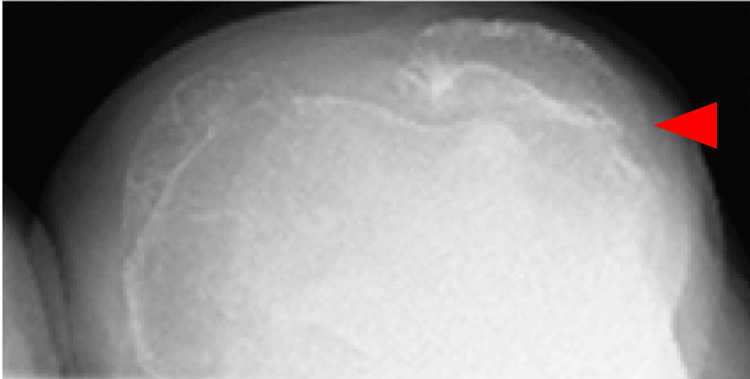
Skyline view of the patella at 30 degrees of knee flexion

**Figure 10:**
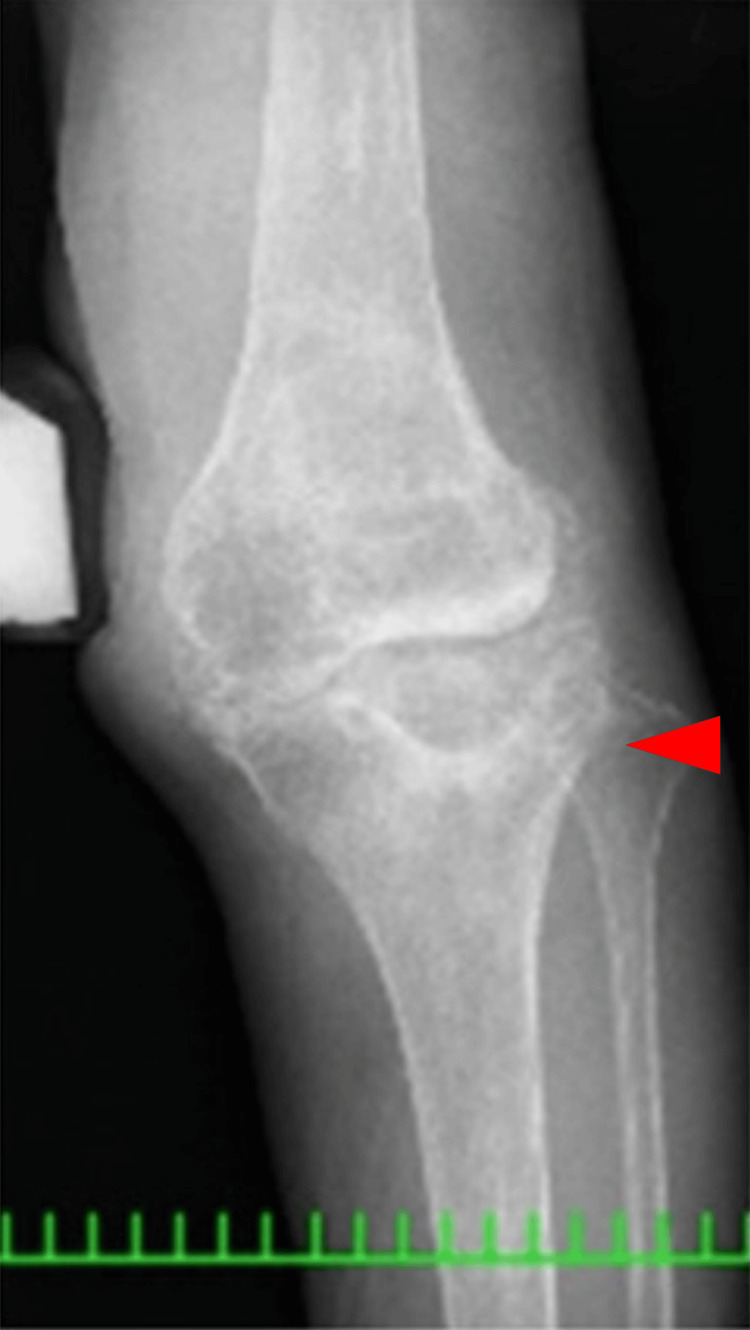
Varus stress image of the knee

**Figure 11:**
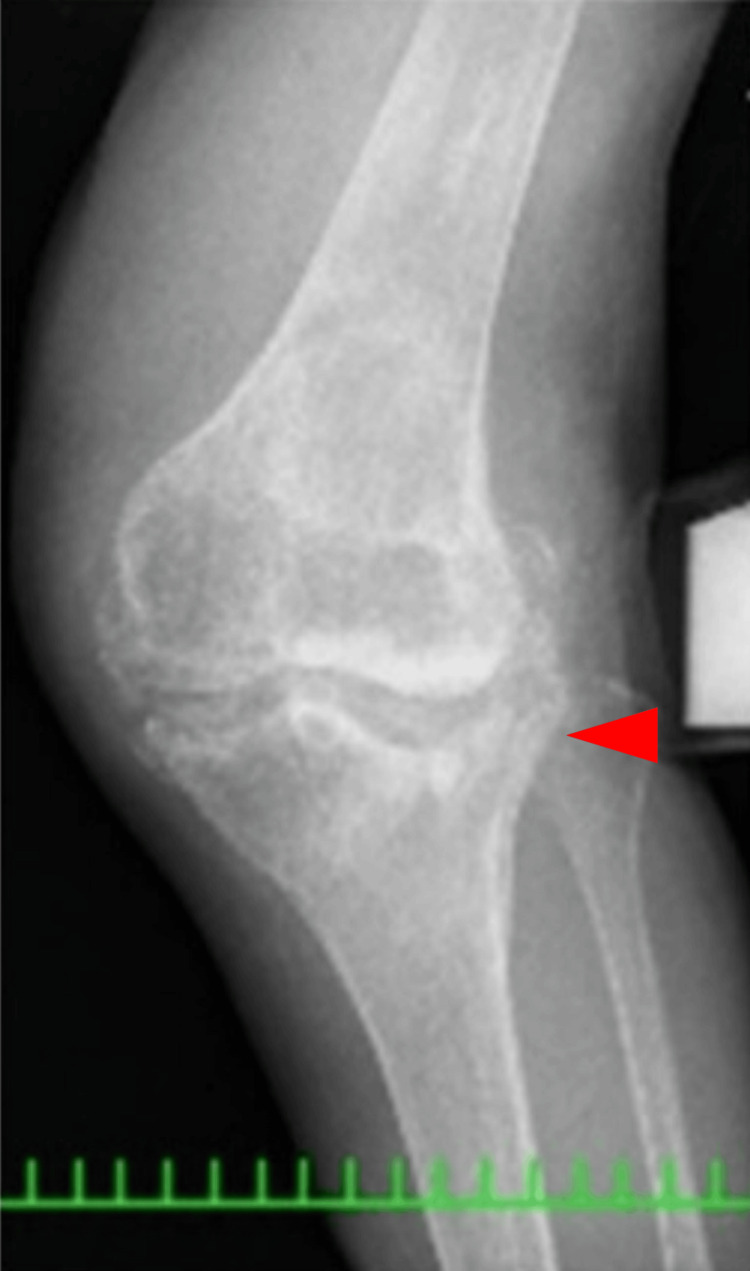
Valgus stress image of the knee

**Figure 12:**
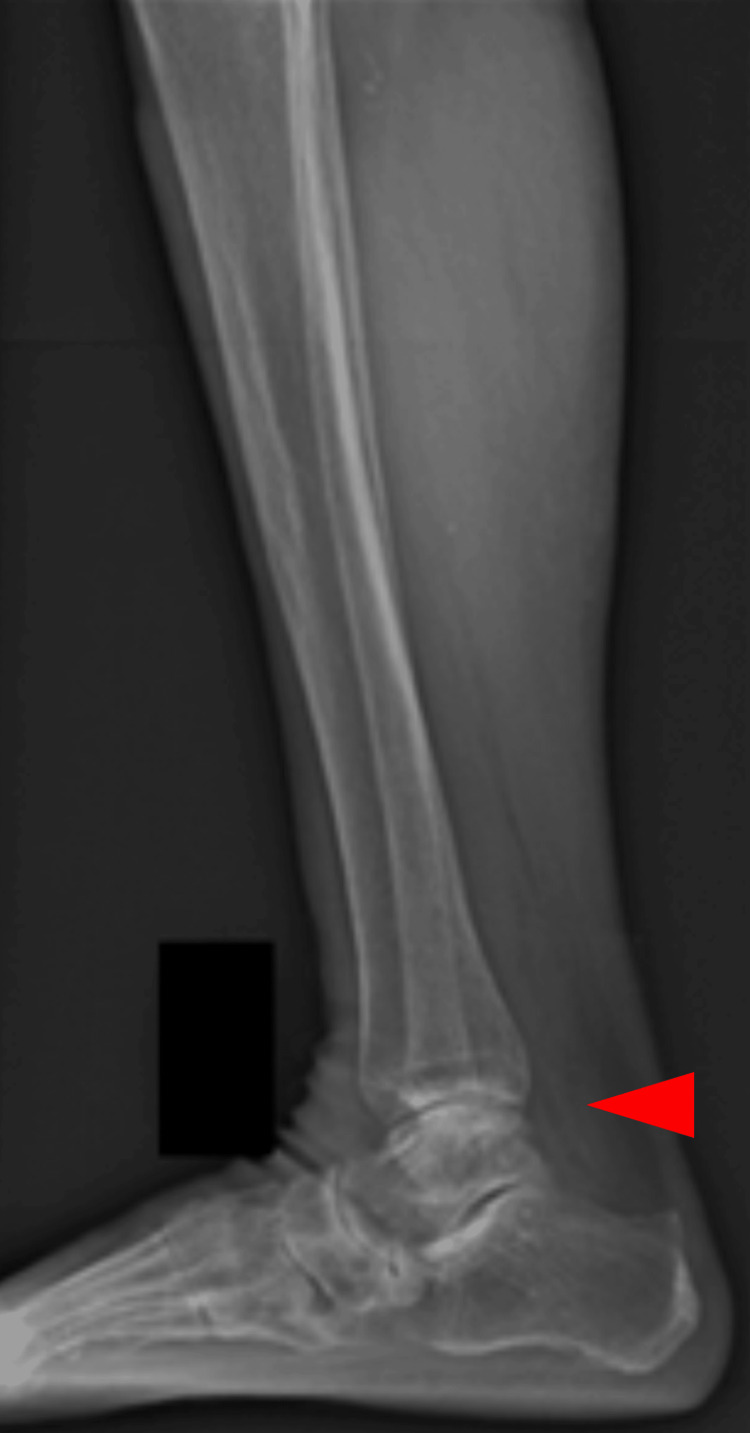
Lateral standing image of the right ankle

**Figure 13:**
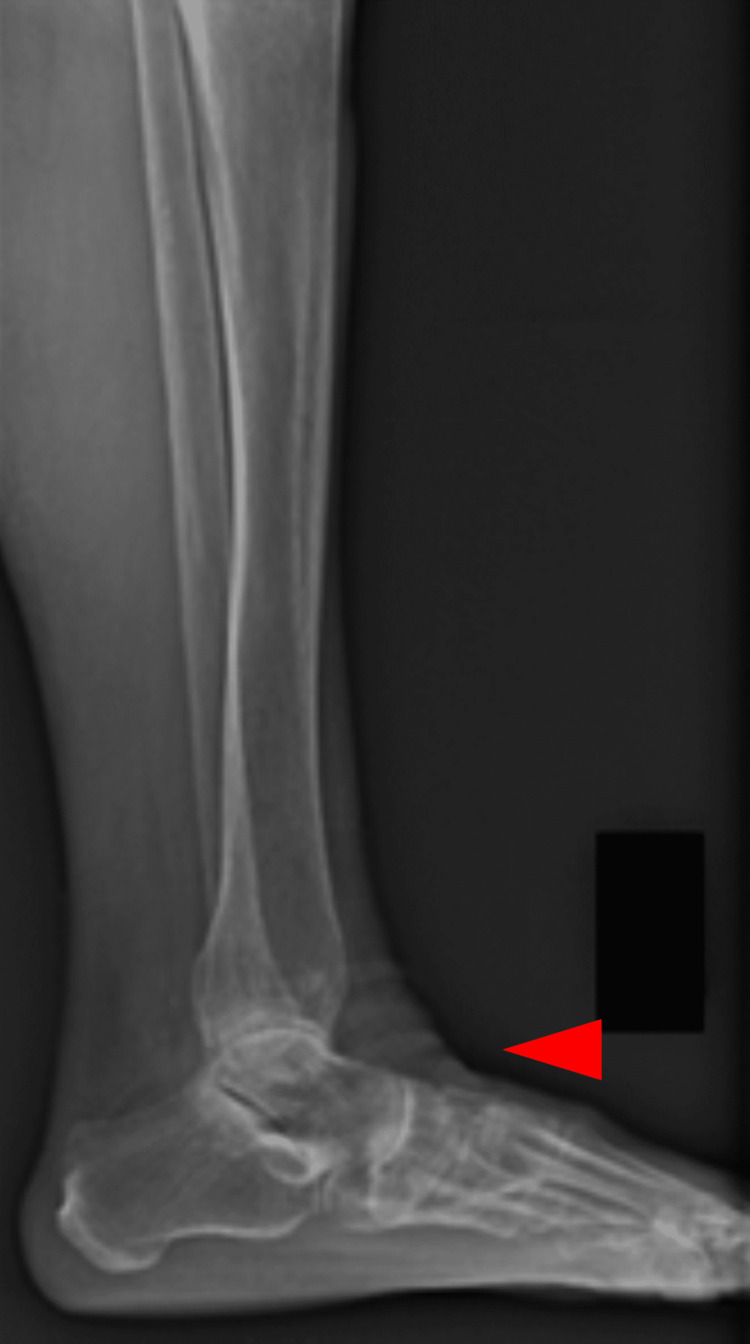
Lateral standing image of the left ankle

Coagulation and syphilis tests were normal. Additionally, reduced nerve conduction velocity was noted. A long leg standing radiograph showed an obvious valgus deformity and destruction of the tibiofemoral joint-the HKA of the left knee was 31 degrees, the LDFA of the left knee was 77 degrees, and the MPTA of the left knee was 81 degrees. A stress radiograph showed a correction of the HKA to -1 degrees with valgus stress. A lateral shift of the patella and hypoplasia of the femoral trochlea were also observed (Figures 7-11). Both ankle joints were painless but observed varus deformity. (Figures 7, 12-13). The KSS clinical score was 13 and KSS functional score was 29.

In line with these findings, the patient was then diagnosed with a Charcot joint secondary to CMT. She was treated with TKA using a NexGen® Rotating Hinge Knee implant. During the surgery, significant deformity of the patellofemoral joint was observed, so patellar resurfacing was performed.

**Figure 14:**
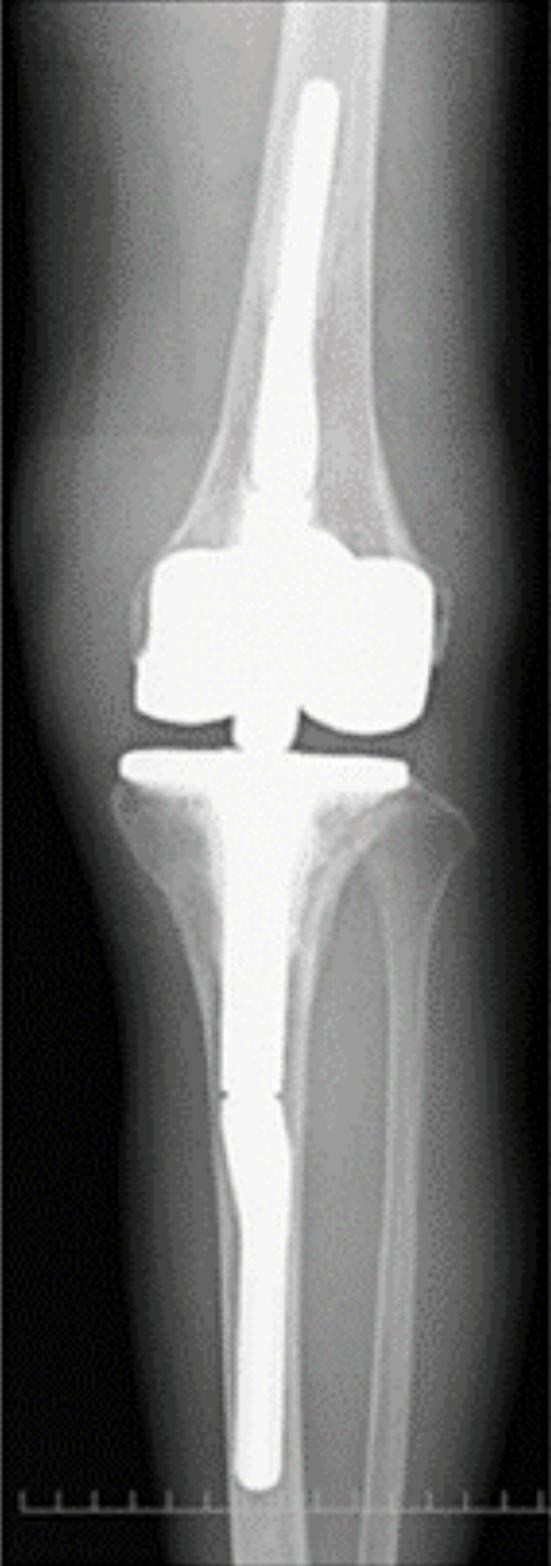
Anteroposterior one-leg standing image of the left knee

**Figure 15:**
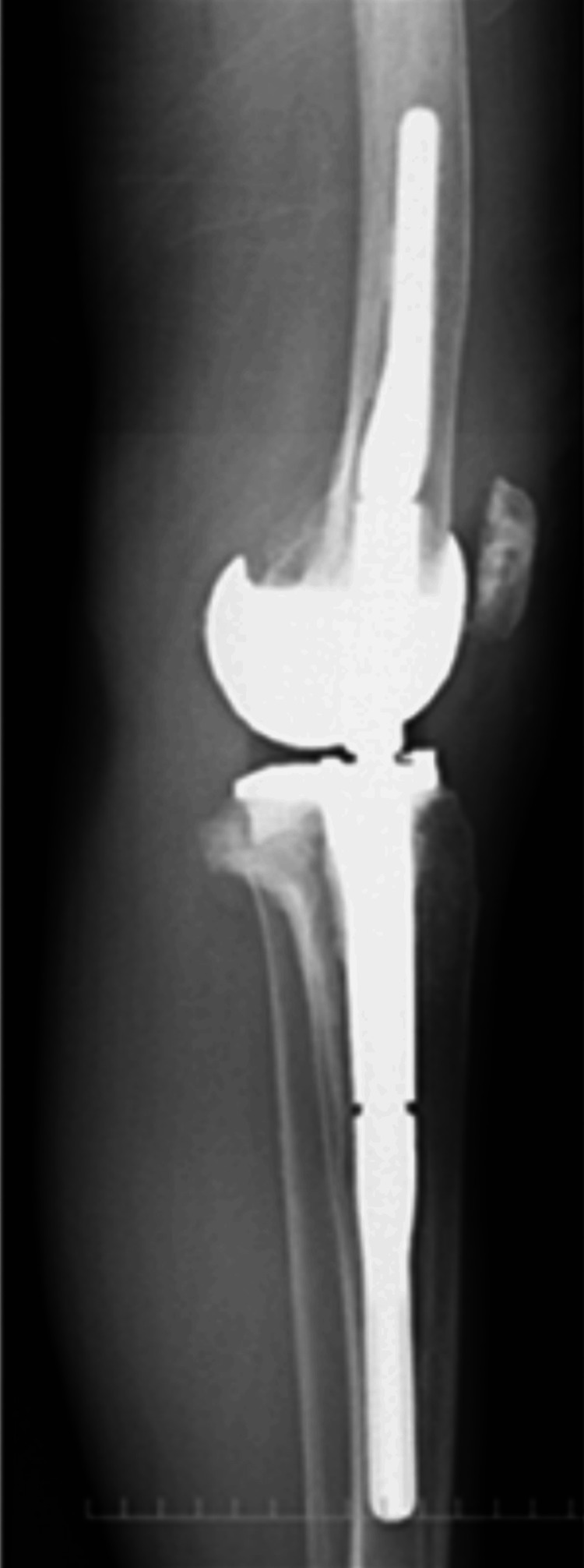
Lateral one-leg standing image of the left knee

**Figure 16:**
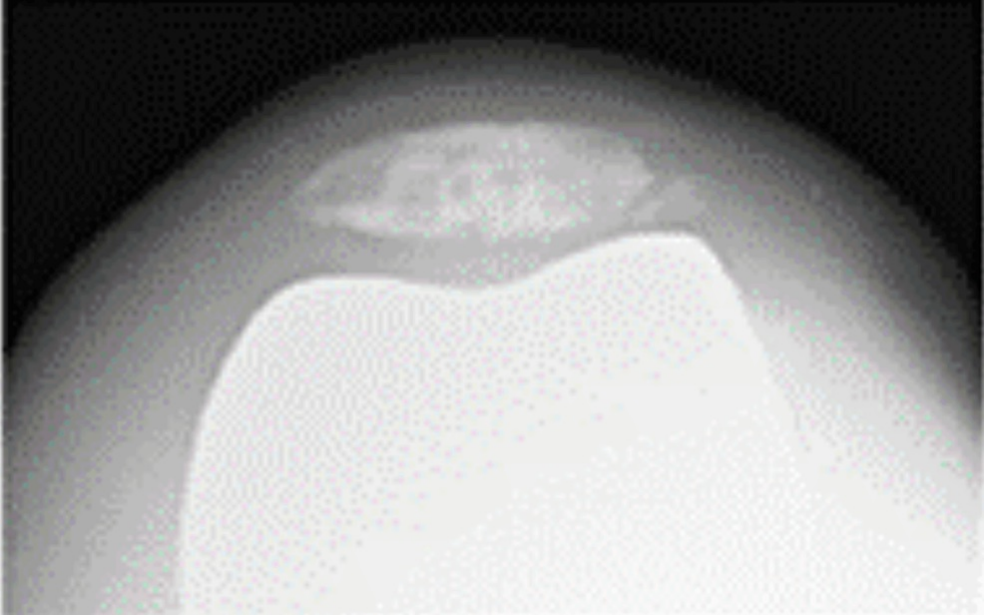
Skyline view of the patella at 30 degrees of knee flexion

One year postoperatively, her left knee range of motion was 100 degrees in flexion and 0 degrees in extension. There was no medial instability, and the patient was able to walk smoothly using a walker. A radiograph showed an HKA of -1, LDFA of 93, MPTA of 95 degrees for the right knee, and no loosening or sinking (Figures 14-16). The KSS clinical score was 89 and the KSS functional score was 29.

## Discussion

We reported good short- to mid-term results of significantly improved KSS clinical scores from constrained TKA for neuropathic arthropathy secondary to CMT. CMT is the most common hereditary disease, and it is reported in 1-4 people per 100,000 in the general population. Its characteristic symptoms include muscle weakness and sensory disturbances of the distal lower limbs. CMT typically develops in childhood or early adulthood, progresses slowly, and results in an unsteady walk [11].

Charcot joint is a type of neurogenic arthropathy and is a relatively rare disease. The etiology and pathophysiology of joint destruction are not well understood. One theory is that Charcot joints involve neurotraumatic (abnormal sensory innervation, repeated micro-traumatic damage, and destruction) and neurovascular (a hypervascular region in the subchondral bone) injuries [6].

A Charcot joint can occur secondary to central or peripheral nerve diseases, such as tabes dorsalis, diabetes mellitus, syringomyelia, congenital insensitivity to pain, or CMT. A Charcot joint was first described as a complication of diabetic neuropathy in 1936, although similar changes can complicate other neuropathies [12]. Previously, tabes dorsalis was the most common cause of Charcot joints; however, diabetic neuropathy is currently the most common cause. Charcot joints have been reported to occur in 0.1-7.5% of patients with diabetes mellitus [13,14]. Charcot joints of the knees in patients with CMT is very rare and there are few reports [3]. It is most frequent in women whose peroneal muscular atrophy has been present for a long time and is severe. Arthropathy affects the ankle (5/22) and foot (3/22); occasionally, the peripheral joints of the upper limb are afflicted [3].

TKA in patients with Charcot joints is technically challenging due to bone defects, soft tissue construction, ligament deficiencies, and muscle weakness. Therefore, a Charcot joint used to be a contraindication for TKA provided the underlying disease was treated [15,16]. Presently, TKA is indicated as a treatment if stem or metal augments are used [5,9,17]. The revision rate has decreased, while the rate of complications such as aseptic loosening, infection, periprosthetic fracture, or implant instability still remains high [5,7-9]. A variety of implants have been used in TKA for Charcot arthropathy, including cruciate retained (CR), posterior stable (PS), Legacy® constrained condylar knee (LCCK), and RHK. The choice of implants is still controversial [18,19]. Unrestrained components (e.g., CR PS) are often not suitable for Charcot arthropathy because they can cause postoperative joint instability due to severe deformity and soft tissue imbalance [4,18]. For this reason, LCCK, which provides good stability with minimal restrictions, is considered the preferred implant for Charcot arthropathy by some surgeons [7, 8]. RHK is indicated in patients presenting with knee hyperextension because excessive restraint can increase the risk of aseptic loosening and periprosthetic fractures [19,20]. A short-term postoperative follow-up after RHK for Charcot joints secondary to CMT and myasthenia gravis revealed improved knee function [10]. One report suggested that patients with Charcot joints and muscle weakness of the lower limbs should undergo RHK to prevent instability post-surgery [9]. Reports on the mediate to long-term results of RHK are as follows. Good intermediate-term results in terms of pain and function have been reported with RHK replacement for revision TKA [21]. Gudnason et al. reported that the 10-year survival rate of a revision RHK was 65.1% for an average of 8.8 months of follow-up [22]. We performed RHK arthroplasty because of the extremely high degree of medial instability and the fact that CMT is a neurological disorder, which raised concerns about the future hyperextension of the knee joint; RHK was used as a precaution. The short to mid-term postoperative follow-up revealed improved knee function and the resolution of all symptoms on the operated side.

Very few studies have reported on the outcome of TKA for Charcot knees in patients with CMT. In our cases, the patients presented with earlier onset of muscle weakness and sensory disturbances of the distal lower limbs, deformity, and significant medial instability of the respective knees. We performed RHK arthroplasty because of the severe medial instability. However, there are several limitations. While deformities of the feet are characteristic in CMT, due to the absence of a radiographic examination of the feet, it was not possible to evaluate the deformity in detail. Although good short to mid-term results were achieved after surgery, careful follow-up is necessary in the future.

## Conclusions

TKA is indicated for the treatment of Charcot joint provided that the underlying disease is controlled. The short to mid-term clinical outcomes of RHK arthroplasty for Charcot joints secondary to CMT were encouraging, although a long-term follow-up is necessary to draw conclusions. RHK arthroplasty can be a viable therapeutic option for the patient population.
